# Total Wake: Natural, Pathological, and Experimental Limits to Sleep Reduction

**DOI:** 10.3389/fnins.2021.643496

**Published:** 2021-04-07

**Authors:** Yuri Panchin, Vladimir M. Kovalzon

**Affiliations:** ^1^Institute for Information Transmission Problems, Russian Academy of Sciences, Moscow, Russia; ^2^Department of Mathematical Methods in Biology, Belozersky Institute, Lomonosov Moscow State University, Moscow, Russia; ^3^Severtsov Institute of Ecology and Evolution, Russian Academy of Sciences, Moscow, Russia

**Keywords:** sleep, non-REM sleep, REM sleep, Morvan syndrome, agrypnia, evolution

## Abstract

Sleep is not considered a pathological state, but it consumes a third of conscious human life. This share is much more than most optimistic life extension forecasts that biotechnologies or experimental and medical interventions can offer. Are there insurmountable physical or biological limitations to reducing the duration of sleep? How far can it be avoided without fatal consequences? What means can reduce the length of sleep? It is widely accepted that sleep is necessary for long-term survival. Here we review the limited yet intriguing evidence that is not consistent with this notion. We concentrate on clinical cases of complete and partial loss of sleep and on human mutations that result in a short sleep phenotype. These observations are supported by new animal studies and are discussed from the perspective of sleep evolution. Two separate hypotheses suggest distinct approaches for remodeling our sleep machinery. If sleep serves an unidentified vital physiological function, this indispensable function has to be identified before “sleep prosthesis” (technical, biological, or chemical) can be developed. If sleep has no vital function, but rather represents a timing mechanism for adaptive inactivity, sleep could be reduced by forging the sleep generation system itself, with no adverse effects.

Dedicated to Michel Valentin Marcel Jouvet (1925–2017)

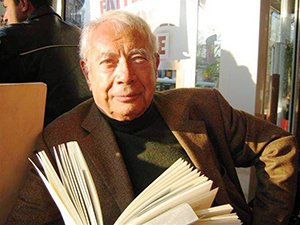

Michel Jouvet, 2005

(Photo by Vera Nezgovorova)

## Introduction: Always Look On The Bright Side Of Wake

This Mini Review was inspired by the striking clinical case (now almost forgotten) of agrypnia (the complete loss of sleep) reported by Michel Jouvet and his colleagues (Fischer-Perroudon et al., 1974) that was brought back to life in his last book *Le sommeil, la conscience et l’éveil* (*Sleep, Consciousness and Wakefulness*) (Jouvet, 2016). Complaints regarding complete insomnia are not unusual for medical somnologists. Yet close examination reveals that such patients actually sleep without realizing it (Rezaie et al., 2018). There are only a few conditions that are believed to result in agrypnia. These conditions are strongly associated with deleterious effects upon the patients’ health and are often presented as arguments for the absolute necessity of human sleep. For instance, fatal familial insomnia (FFI) is a prion disease that results in severe suffering and death within a few months to a few years (Lugaresi et al., 1986). There are a number of publications insisting that a sufficient duration of sleep is essential for high cognitive performance during wakefulness and prevents adverse and even lethal outcomes. In contrast to this concept, Jouvet describes a patient that had not slept for at least 4 months with no disturbance of either attention or memory. Here we discuss this and other new and old publications that show extreme sleep reduction in humans and animals.

## Life Without Sleep

The author’s preface of Jouvet’s last book starts with this case description: “There is such pathology as Morvan’s disease, in which quasi-wakefulness, which lasted 3,000 h (more precisely, 2,880), or 4 months, was not accompanied by sleep rebound, since the sleep generation system itself was disturbed.” Throughout this period the patient was under continuous polysomnographic control, so his agrypnia was confirmed objectively. Jouvet conclude that “… slow wave (NREM) and paradoxical (REM) sleep are not necessary for life (at least for 4–5 months for the first and about 8 months for the second), and we cannot consider their suppression to be the cause of any serious disorders in the body. A person who had lack of sleep and dreams for 4 months, of which there are only a few minutes of nightly hallucinations, can turn out to read newspapers during the day, make plans, play cards and win, and at the same time lie on the bed in the dark all night without sleep! In conclusion, we admit: this observation makes most theories about the functions of sleep and paradoxical (REM) sleep obsolete at once, but offers nothing else” (Jouvet, 2016).

Morvan syndrome is a very rare disease that varies greatly in prevalence and magnitude of key symptom manifestation including severe insomnia. Approximately 90% of cases spontaneously go into remission, while the other 10% of cases lead to death. Only approximately 20 cases of Morvan syndrome have been reported with different degrees of sleep disorder (Liguori et al., 2001; Abgrall et al., 2015; Maskery et al., 2016; Vale et al., 2017) and unfortunately they do not match Jouvet’s vivid description.

The etiology of this syndrome is not understood. It was reported that the syndrome is associated with the production of autoantibodies to voltage-gated potassium channel complexes (VGKCs) (Masood and Sitammagari, 2020). Although VGKC antibodies may have an effect on the peripheral nervous system, causing neuromyotonia (Löscher et al., 2004), it is not clear if they may cross the blood–brain barrier and act centrally. At the same time, voltage-gated potassium channels are implicated in sleep–wake regulation in mice and *Drosophila* (Douglas et al., 2007; Cirelli, 2009; Donlea et al., 2017; Yoshida et al., 2018).

Another case of surviving without sleep came from fruit fly studies. In *Drosophila melanogaster*, natural inactivity states are commonly defined as “sleep” (Funato, 2020). *Drosophila* locomotor inactivity lasting at least 5 min shares behavioral hallmarks of sleep in humans such as specific posture, increased arousal threshold, reversibility, and homeostatic regulation, and when following rest (“sleep”) deprivation, flies showed an increase in consecutive “sleep” (Hendricks et al., 2000; Shaw et al., 2000; Funato, 2020). Some studies have reported that sleep deprivation in flies could be lethal or decrease lifespan (Shaw et al., 2002; Ly et al., 2018; Vaccaro et al., 2020).

Nevertheless, sleepless flies have been described (Geissmann et al., 2019). With machine learning-based video-tracking technology it is possible to recognize and attribute to wakefulness not only individual locomotor activity (walking or flying) but also other active behaviors such as egg laying, grooming, and feeding. Using this advanced technique, the authors analyzed the behavior of 1,366 flies from the Canton-S strain that are commonly used as “wild types.” Screening revealed a wide range of phenotypes including virtually sleepless flies. While the average duration of daily sleep was 10 h and 20 min in males and 5 h in females, 6% of female flies slept less than 72 min a day, and three flies slept an average of 15, 14, and 4 min a day, respectively. The authors also re-examined sleep deprivation effects in a lifelong experiment using a closed-loop sleep deprivation device. Although this procedure led to almost complete sleep deprivation, no considerable effects on fly survival were noticed. The value and importance of fly sleep homeostasis was also challenged in this study by demonstrating that 10 days of chronic sleep deprivation in male flies produces sleep rebound of the same value as only one night of acute sleep restriction. Experiments employing artificial selection for high and low sleep duration over 13 generations in *Drosophila* allowed the extension of sleep duration to almost 18 h in long sleepers and reduced it to approximately 3 h in the short sleepers (Harbison et al., 2017). In this experiment, all flies (in total 15,607) were monitored during 4-day tests and no sleep was detected at all in 15 flies. This study does not provide any specific information regarding the altered behavior of the flies with lack of sleep and short-sleeping flies compared to normally sleeping and long-sleeping flies. However, the authors point out that any physiological consequences of excessively long or short sleep did not manifest themselves either in life expectancy or in vitality during ontogenesis. If the state of inactivity in flies is indeed evolutionally related to human sleep, then it is not mandatory; some individuals may not need it at all, and artificial selection can dramatically reduce sleep-like states in a population.

## Life Without Rem Sleep

Mammalian sleep alternates between two distinct modes: non-rapid eye movement (NREM) and rapid eye movement (REM) sleep. REM sleep represents a smaller portion of total sleep time and is associated with desynchronized and fast brain waves, eye movements, and loss of muscle tone. It is a matter of dispute as to which form of sleep, REM or non-REM, is more important (Siegel, 2009; Blumberg et al., 2020).

An extraordinary REM sleep anomaly was identified in a patient with shrapnel wound that damaged his brainstem, thalamus, and cerebellum (Lavie et al., 1984). Upon recovery he completed his education and became a lawyer and a painter (Magidov et al., 2018). Years later, polysomnographic recordings revealed that he had very short REM sleep (an average of 6 min instead of the normal 1.5–2 h). Thus, a person who went (almost) without REM sleep practically all his adult life did not show any disadvantages that could be attributed to its absence. Peretz Lavie stated that “he is probably the most normal person I know and one of the most successful ones” (Vertes and Siegel, 2005).

Other evidence for the possibility of “living without REM sleep” is provided by psychopharmacology. Most antidepressant drugs (MAO inhibitors, tricyclics, and selective serotonin reuptake inhibitors) profoundly suppress REM sleep (Riemann et al., 2020). Of course, not all antidepressants cause REM sleep suppression. For example, the atypical antidepressant nefazodone, the serotonin reuptake inhibitor trazodone, and the tricyclic antidepressant trimipramine not only fail to suppress REM sleep but may even restore disturbed sleep patterns in depressed patients (Wichniak et al., 2017; Riemann et al., 2020). Nevertheless, most antidepressants, and especially MAO inhibitors, very strongly suppress REM sleep, up to its complete disappearance. Many patients have shown polysomnographically confirmed REM sleep loss for years without any noticeable cognitive impairment (Siegel, 2001).

In a meticulous study of the role of brain acetylcholine in sleep, Niwa et al. (2018) engineered a mouse strain that underwent almost no REM sleep. The authors conducted exhaustive analyses of sleep phenotypes in all acetylcholine receptor gene-knockout (KO) mice. KO of two muscarinic acetylcholine receptors, Chrm1 and Chrm3, showed noticeable effects on sleep. Yet the most outstanding effect in this study was shown in Chrm1/3 double-KO mice, with almost undetectable REM sleep and reduced NREM sleep. At the same time, the authors did not find any obvious abnormalities in Chrm1/3 double-KO mice resembling those found after sleep deprivation (Banks et al., 2017).

Natural cases of life without REM sleep occur in dolphins (Lyamin et al., 2008). One explanation for REM loss in these aquatic mammals is given by Markus H. Schmidt, according to the energy allocation function of his sleep model. He suggested that the high thermal conductance of water is not compatible with the loss of thermoregulation in REM sleep (Schmidt, 2014).

## Life With Reduced Sleep Duration

Sleep duration varies greatly among individuals partially due to genetic factors. Several human mutations strongly affecting this phenotype were recently identified in families that require less than normal sleep time. People with familial natural short sleep (FNSS) sleep for only 4–6 h per night while feeling well rested and show no evident disorders usually associated with chronically restricted sleep.

A mutation in the basic helix–loop–helix transcription factor DEC2 (hDEC2-P385R) was found to be associated with the human short sleep phenotype in two affected individuals from one family with extremely early wake-up times and habitually short (6.25 h on average) total sleep time. Transgenic mice carrying this mutation showed a reduced amount of both NREM and REM sleep (He et al., 2009).

Another autosomal dominant allele from one family with naturally short sleep was identified in the metabotropic beta-1 adrenergic receptor (ADRB1) that belongs to the family of 7TM GPCR proteins. Carriers of this mutation have a lifelong tendency to sleep only 4–6 h per night and feel well rested. The introduction of this mutation to mice resulted in a shorter sleep phenotype with approximately 55 min shorter total sleep time compared to wild-type mice, which affected both non-REM and REM sleep (Shi et al., 2019).

Recently, the natural short sleep phenotype in humans was shown to be associated with a missense mutation in the G protein-coupled neuropeptide S receptor 1 (NPSR1-Y206H). This mutation was identified by the same group that identified the two other abovementioned genes associated with short sleep (Xing et al., 2019). Subjects from the family in which the mutation was found reported only 5.5 and 4.3 h of regular optimal sleep duration. Engineered genetically modified mice with a homologous tyrosine to histidine substitution also display reduced sleep time (by approximately 71 min). This reduction primarily contributed to NREM sleep. The data are consistent with previous reports on neuropeptide S action on sleep behavior in mice. In humans, the homozygous mutation N107I in the extracellular loops of NPSR1 was also associated with slightly reduced sleep time (Gottlieb et al., 2007; Spada et al., 2014). Importantly, Npsr1 knockout (Npsr1^–/–^) mice show no difference in sleep duration compared to the wild type, whereas NPSR1-Y206H is a gain-of-function mutation (Xing et al., 2019).

A massive electroencephalogram/electromyogram-based screen of 8,000 randomly mutagenized mice identified two dominant mutations that affect sleep and wakefulness (Funato et al., 2016). One of these was the splicing, exon-skipping, mutation *Sleepy* in the Sik3 protein kinase gene that causes a decrease of approximately a quarter of total wake time compared to wild-type littermates. Another gain-of-function mutation, *Dreamless*, in the sodium leak channel Nalcn was identified by the same screen. It caused a 44% reduction in REM sleep time. These results confirmed that the sleep–wakefulness cycle can be widely genetically modified.

Natural temporary sleep loss is commonly seen during specific periods in the life cycle of some bird species, including the mating, migration, and birthing seasons. These examples include migrating sandpipers (Lesku et al., 2012) and Galapagos frigate birds during rearing of their young (Rattenborg et al., 2016). It appears that this temporary sleep loss is not harmful, yet data are limited to only two species, and potential consequences have not been ruled out.

Of particular interest is the organization of alternating one-hemispheric slow-wave sleep, which is lifelong in dolphins (Lyamin et al., 2008) and temporary, depending on the environment, in eared seals (Lyamin et al., 2017). However, discussion of this issue is not possible within the framework of this brief overview.

## Sleep Evolution

Sleep is important and widespread. Sleep doubtlessly emerged by natural selection; however, there are two possible distinct routes for its evolution. Here, we term them the indispensable sleep scenario (ISS) and the adaptive inactivity scenario (AIS). Under the ISS, sleep meets the immediate clear and present needs of an individual organism and serves some vital function(s) that are accompanied by periodic discontinuation of activity, while this dormancy itself is not beneficial or even compromises fitness. This view is widespread in the scientific community (Brown et al., 2012). Alternatively, periodical inactivity itself is evolutionarily adaptive for individuals and is maintained by natural selection. This is why it depends on behavioral and environmental factors and can vary greatly from complete absence to extensive values. This view is most strongly advocated by Jerome M. Siegel (2009). If sleep is adaptive and does not serve an unknown vital function, then from an evolutionary prospective, sleepless animals would be eliminated from the population not because they die from sleep absence but because extra wake time makes them more vulnerable in the wild. Assuming that the “no bustling” strategy is adaptive, sleep-like behaviors could evolutionarily arise in different groups of organisms independently and more than once.

The energy allocation function of a sleep model is also not focused on a single vital function emphasizing the general energy-saving benefits of optimization of temporal energy distribution in the sleep–wake cycle and does not predict that all animals sleep (Schmidt, 2014; Schmidt et al., 2017; Latifi et al., 2018).

Playing possum or thanatosis behavior, torpor, and hibernation are examples of adaptive inactivity states distinct from sleep (Schmidt, 2014; Jastroch et al., 2016). Is quiet wakefulness a substitute for sleep? Wakefulness behavior is controlled by a different sensory stimulus resulting, for instance, in the fight-or-flight response or an internal urge for sex or food. Quiet wake that may compete with sleep to produce evolutionarily adaptive periodical inactivity requires radical rewiring of existing wake reflexes. Sleeping could be a more simple evolutionary innovation to achieve this adaptation.

The dopaminergic ultradian oscillator (Blum et al., 2014) is another example of the mechanism that modulates animal activity independently from both sleep and the circadian pacemaker. It could be implied that the biological role of sleep that evolved under the AIS was evolutionarily enhanced by synchronization and linkage of some physiological functions to sleep–wake timing, similar to circadian cycling that adjusts various physiological parameters such as melatonin secretion, core body temperature, and cortisol levels according to daily rhythms.

How can we tell between these different evolutionary trends? In the first case, we expect the existence of some vital organismal function that could be completed only during sleep. Do we know of any such functions, and is there any evidence that their existence is vital? Reports of the lethal effects of sleep deprivation support the ISS. However, is it true that sleep loss is lethal?

## Sleep Functions

Different bodily functions are considered to benefit from sleep, aside from adaptive inactivity. These includes physical rest, neurogenesis, memory, immune system regulation, and brain cleansing, although there is no explanation for why they can only be performed during sleep (Siegel, 2009). Although brain cleansing is not indispensable for survival, there is reasonable evidence linking it with NREM sleep. The studies of Nedergaard and colleagues showed that “flushing” the brain of toxic waste occurs during NREM sleep (Xie et al., 2013; Ding et al., 2016). A recent study (Fultz et al., 2019) demonstrated that synchronous slow-wave sleep activity causes massive dilation of blood vessels and an increase in the total volume of blood in the brain parenchyma *via* the neurovascular unit. Since total brain volume is constant, the change in blood volume facilitates the circulation of cerebrospinal fluid. Possibly in the absence of the real brain-confined lymphatics, wake-incompatible synchronous slow-wave neuronal activity drainage mechanisms promote the divergence of the opposing wake–sleep states in evolution.

## Death Without Sleep

Probably the most powerful argument in favor of sleep serving a vital function is the observation that chronic sleep restriction leads to death. In humans, FFI, according to its name, is accompanied by sleep loss and leads to death. Yet, this disease manifests in severe neuronal degeneration and reveals other deleterious symptoms. So it is not clear if death in FFI is caused by sleeplessness or other symptoms. Mutations in the same prion protein gene, PRNP, that evokes FFI also cause Creutzfeldt–Jakob disease (CJD), which is also deadly, but without agrypnia (Zarranz et al., 2005). FFI and CJD share the same mutation at codon 178 of the prion protein gene. However, FFI is also consistently linked to the presence of a methionine codon at position 129, whereas in CJD that position encodes valine. It is very interesting if this subtle difference, which by itself harmless, can redirect the syndrome to manifest in sleeplessness. Knockin mice expressing human FFI-specific PRNP mutations develop some abnormalities reminiscent of the human disease, but not agrypnia (Jackson et al., 2009; Bouybayoune et al., 2015). Likewise, there is no conclusive evidence that Morvan syndrome death outcomes are caused by sleep loss.

The hypothesis that sleep is critical for survival and that total sleep deprivation (TSD) is lethal has been explored in animals. For such studies it is crucial to exclude factors others than sleep loss affecting the animals. It is generally agreed that the historical experiments in this field (Bentivoglio and Grassi-Zucconi, 1997; Kovalzon, 2009) lacked proper controls. Allan Rechtschaffen and colleagues made a special effort to maintain maximum symmetry between experimental and control rats, so that they were exposed to identical stimuli. They reported lethal consequences of long-term TSD and REM sleep deprivation and concluded that sleep does indeed serve an unidentified vital physiological function (Rechtschaffen et al., 1983). This remarkable result was followed by more publications (Rechtschaffen and Bergmann, 1995; Everson and Toth, 2000). However, these experiments originated mostly from the same research groups, and the results were not replicated in other mammals. The same method when applied to pigeons did not show TSD to be lethal (Newman et al., 2008). Although data indicating that chronic sleep loss is fatal should be taken into account, alternative interpretations cannot be entirely excluded and warrant further investigation.

## Conclusion

Is it practically possible to achieve harmless substantial sleep reduction by neuroengineering or by other means? Two distinct hypotheses (ISS and AIS) of sleep function and evolution apply different limitations to this goal. If sleep serves an unidentified vital physiological function, this function has to be elucidated before developing some kind of “sleep prosthesis” (technical, biological, or chemical). For instance, if the vital function of sleep is to “flush” harmful waste from the brain, it could be theoretically achieved by some artificial method or device.

Alternatively, the sleep drive can be viewed as a feature that could be modified with no harmful consequences. In comparing Morvan’s disease, agrypnia, to experimental sleep deprivation in humans (Gulevich et al., 1966; Kollar et al., 1969), Jouvet (2016) specified that “In the first case, the patient wanted to sleep and was constantly looking for sleep, but could not fall asleep even after 4 months of insomnia. In the second case, on the contrary, the subjects constantly struggled with sleep and after 120, 200, and 264 sleepless hours fell from fatigue, demonstrating a long “rebound” of sleep”. If it does turn out that sleep has no vital function, but represents a timing mechanism, progress in neuroscience and neuroengineering may allow us to reduce sleep by harmlessly forging the sleep generation system itself. Even if reviewing data on healthy sleep reduction does not provide “total wakefulness,” it may help us deal with undesirable and even dangerous conditions, such as local sleep and microsleep that are associated with significant cognitive impairments and not recognized by the subject (D’Ambrosio et al., 2019).

## Author Contributions

YP and VK contributed to the conception, writing, and revising of this manuscript. Both authors contributed to the article and approved the submitted version.

## Conflict of Interest

The authors declare that the research was conducted in the absence of any commercial or financial relationships that could be construed as a potential conflict of interest.
